# Portable on-chip colorimetric biosensing platform integrated with a smartphone for label/PCR-free detection of *Cryptosporidium* RNA

**DOI:** 10.1038/s41598-021-02580-w

**Published:** 2021-12-01

**Authors:** George S. Luka, Ephraim Nowak, Quin Robert Toyata, Nishat Tasnim, Homayoun Najjaran, Mina Hoorfar

**Affiliations:** School of Engineering, Faculty of Applied Science, The University of British Columbia, Kelowna, BC V1V 1V7 Canada

**Keywords:** Engineering, Mechanical engineering, DNA nanotechnology, Nanoscale devices, Nanoscale materials, Other nanotechnology, Techniques and instrumentation

## Abstract

*Cryptosporidium*, a protozoan pathogen, is a leading threat to public health and the economy. Herein, we report the development of a portable, colorimetric biosensing platform for the sensitive, selective and label/PCR-free detection of *Cryptosporidium* RNA using oligonucleotides modified gold nanoparticles (AuNPs). A pair of specific thiolated oligonucleotides, complementary to adjacent sequences on *Cryptosporidium* RNA, were attached to AuNPs. The need for expensive laboratory-based equipment was eliminated by performing the colorimetric assay on a micro-fabricated chip in a 3D-printed holder assembly. A smartphone camera was used to capture an image of the color change for quantitative analysis. The detection was based on the aggregation of the gold nanoparticles due to the hybridization between the complementary *Cryptosporidium* RNA and the oligonucleotides immobilized on the AuNPs surface. In the complementary RNA’s presence, a distinctive color change of the AuNPs (from red to blue) was observed by the naked eye. However, in the presence of non-complementary RNA, no color change was observed. The sensing platform showed wide linear responses between 5 and 100 µM with a low detection limit of 5 µM of *Cryptosporidium* RNA. Additionally, the sensor developed here can provide information about different *Cryptosporidium* species present in water resources. This cost-effective, easy-to-use, portable and smartphone integrated on-chip colorimetric biosensor has great potential to be used for real-time and portable POC pathogen monitoring and molecular diagnostics.

## Introduction

*Cryptosporidium* spp. is a major intestinal protozoan pathogen in adults, children and numerous animal species worldwide. Recently, cryptosporidiosis, an infection caused by *Cryptosporidium*, has become a global threat to public health and the economy^1^. Moreover, *Cryptosporidium* contamination has increased the challenge to deliver safe drinking water in developing and developed countries^2–4^. The pathogen can cause death in children and immuno-compromised individuals and severe cryptosporidiosis in healthy adults^5,6^.

Several techniques have been used to detect *Cryptosporidium* oocysts in water samples, including fluorescence microscopy-based methods (e.g. Environmental Protection Agency (EPA) 1623)^7^, immunoassays^8,9^ and molecular techniques^10,11^. These methods are time-consuming, insufficient, expensive and require well-trained personnel. In particular, immunological and molecular techniques require extensive sample preparation, including labelling with expensive fluorescence labels, amplification and sophisticated lab-based equipment for detection and data analysis^12^. These challenges make this method unsuitable for on-site detection and the fast-decision-making process^13,14^. Therefore, it is necessary to develop a simple, user-friendly, rapid, cost-effective, label/PCR-free and sensitive method for on-site detection of *Cryptosporidium* in water samples.

Recently, colorimetric detection based on inorganic nanomaterials has attracted great attention due to their simplicity, cost-effectiveness, and distinct color variation associated with their morphology or size change^15–17^. Moreover, colorimetric assays can be visualized with the naked eye without the need for sophisticated instruments or tedious training^18^. Gold nanoparticles (AuNP) colorimetric assays are of particular interest because of their unique features, including their catalytic properties and controllable sizes^19^. More importantly, they are easily synthesizable and modified with several thiolated molecular recognition elements^20^. Furthermore, the unique localized surface plasmon resonance (LSPR) properties of AuNPs allow them to have different colors based on their size^21^. For example, AuNPs with 20 nm have a red color, and their LSPR can be measured at a wavelength of 520 nm^21,22^. Colloidal AuNPs are negatively charged and hence repel each other. The repulsion between the particles allows them to stay colloidal and maintain their red color. However, when salt such as NaCl is added, these negative charges are neutralized (masked), resulting in the aggregation of AuNPs. Aggregation results in a decrease in the distance between the particles and an increase in their size. This results in a change in color from red to blue due to a large shift of the LSPR peak (absorption at a longer wavelength)^23^.

In DNA biosensing, gold nanoparticles are usually modified with a biological recognition element such as a complementary oligonucleotide specific and selective to the target DNA or RNA. The hybridization between the modified AuNPs and the target DNA or RNA in the sample results in a change in color from red to blue. The color change is proportional to the analyte concentration in the sample and can be qualitatively detected by the naked eyes^24^. Due to these features, AuNPs have led to a new generation of biosensors with high stability, selectivity and sensitivity.

Recently, portability has become an increasingly important goal for developing sensing platforms for many applications^25^. On-chip biosensors are one of the most exploited techniques used to produce cost-effective and portable miniaturized sensing platforms^26^. A typical on-chip-based biosensor consists of a recognition element (such as antibodies^27^, nucleic acids^28^, enzymes^29,30^, aptamers^31^ or whole cells^32^), transducer, and detector^33^. The recognition element dictates the biosensing platform specificity and selectivity^34^, while the transducer transforms the response produced from a biorecognition event to a measurable signal^35^. These platforms can be advantageous in a wide range of applications, including food safety^36^, diagnostics^37^, security and defense^38,39^, and environmental monitoring^40^. This is due to their cost-effectiveness, disposability, simplicity, low power requirements and sample-reagent usage^40^. However, most colorimetric detection methods still need advanced and expensive equipment to read and analyze the results^41,42^. Therefore, the applicability of using these techniques for on-site detection and point-of-care (POC) is still limited^43,44^.

In the last decade, smartphones with internal memory storage, touchscreen displays, internet connectivity (enabling data exchange wirelessly over the internet), advanced compute power, and high-resolution cameras have gained significant market acceptance for POC applications^45^. Given the iniquitousness of smartphone access, smartphone-integrated biosensing platforms can serve as a accessible and low-cost solutions for routine (daily) testing without requiring highly-trained personnel and bulky, expensive and complicated laboratory instrumentations such as spectrophotometers and microscopes, especially in resource-limited countries and rural areas^46,47^. As an example, Breslauer et al*.*^48^ developed a mobile phone-mounted light microscope with 350×magnification and demonstrated its potential for global health applications. Smartphone-based image capture and analysis systems have also been applied for monitoring immunoassays such as colorimetric and fluorescence assays^49,50^, DNA diagnostics^51^, label-free detection of pathogens^52^, quantum-dots labelling^53,54^ and fluorescence microscopy^54,55^. Smartphones have also been used as a spectrophotometer with a wavelength resolution of 5 nm^56^. These advantages have led to increasing research and industrial interest in using smartphones to detect analytes of environmental^57–59^, food safety^60,61^, personal health^62^, and biomedical^50,54,63,64^ applications. This has led to integrating smartphones as smart detectors, signal inducers, and data processors with the on-chip-based biosensing technology for rapid, cost-effective, easy-to-use, real-time and POC diagnostics^65–68^. An additional attachment or cradle is used with these integrations to mount the smartphone and other components^69–72^. These integrated systems have allowed many analyses to be performed remotely outside the laboratory (at the point of need)^56,73,74^.

**Figure 1:**
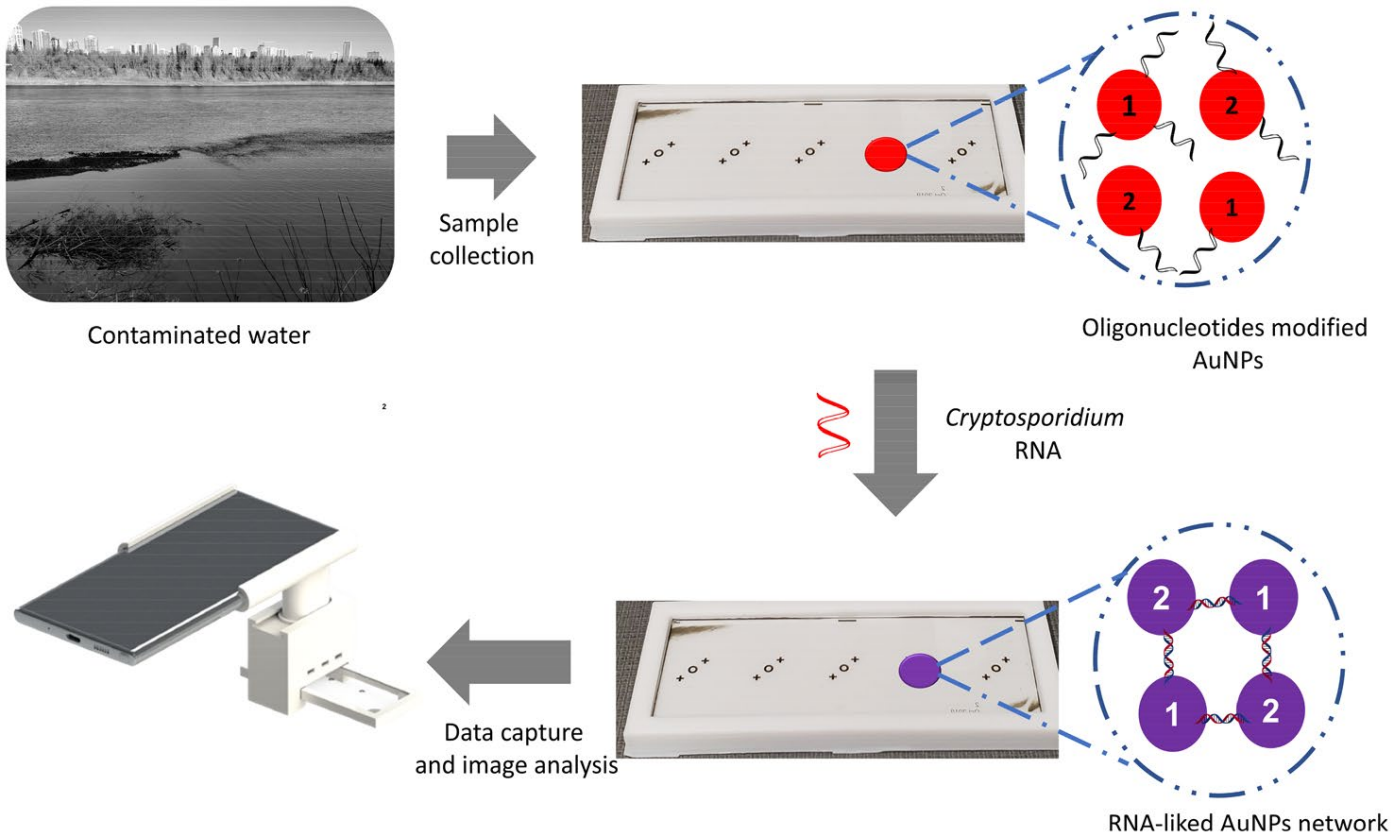
Schematic illustration of label/PCR-free sensing platform.

This research describes the development of a simple, cost-effective, easy to fabricate, sensitive and portable on-chip-based colorimetric biosensor for the direct and label/PCR-free detection of *Cryptosporidium* RNA. Compared to DNA, RNA is shorter and allows for the direct detection of a single mismatch or mutation without amplification (PCR). Hence, RNA was used as the target analyte in this project^75^. The colorimetric sensing mechanism is based on two sets of modified AuNPs with specific oligonucleotides complementary to adjacent sequences on *Cryptosporidium* RNA. Upon adding *Cryptosporidium* RNA into the modified AuNPs solution, hybridization between two complementary sets of oligonucleotides immobilized on the AuNPs and the RNA occurs, forming a network of oligonucleotide-AuNPs. The aggregation of AuNPs results in a colorimetric shift due to the change in LSPR due to a reduction in the distance between the AuNPs and hence an increase in the size of the AuNPs. The outcome is a visible color change of the AuNPs from red to blue, observed and recorded within 5 min (Fig. 1). The colorimetric assay was performed on a fabricated chip integrated with a 3D-printed holder assembly to facilitate its applicability for on-site detection and POC applications. A smartphone in a 3D-printed holder assembly was used to measure the color change due to the target analyte's presence (*Cryptosporidium* RNA). A smartphone was chosen based on its lightweight, portability, on-site image analysis, and data transmission capability. All these advantages are suitable for in-field detection and POC applications. The sensing platform in this research has provided an exclusive sensitivity, selectivity and portability towards the detection of *Cryptosporidium* RNA: a 5 µM of *Cryptosporidium* RNA spiked in water samples can be detected via the developed on-chip colorimetric assay in 30 min, reducing the turnaround detection time following RNA extraction from contaminated sites. The results generated by the developed sensing platform were in good agreement with those generated by the conventional spectroscopic techniques. All these confirm the developed sensing platform combined with the 3D-printed holder assembly integrated with a smartphone could directly detect different *Cryptosporidium* RNA concentrations in a spiked sample without DNA amplification. Furthermore, the on-chip sensing platform developed in this research showed the sensor's capability to detect *Cryptosporidium* and distinguish between its different species. This capability is crucial to allow appropriate risk assessments.

## Materials

Gold nanoparticles (AuNPs) with a diameter of 20 nm were purchased from Cytodiagnostics (Canada). According to the manufacturer, AuNPs were synthesized by citrate reduction, and thus they have negatively charged citrate ions adsorbed on their surface, which enhance their stabilization via electrostatic repulsion. Two sets of thiolated-oligonucleotides capturing probes and *Cryptosporidium* RNA were designed and purchased from Integrated DNA Technologies (IDT, Canada) (Table 1). Sodium phosphate monobasic monohydrate, sodium phosphate dibasic, DL-Dithiothreitol (DTT), sodium azide (NaN_3_), dextran sulfate, formamide, sodium chloride and sodium dodecyl sulfate were purchased from Sigma-Aldrich (Canada). Illustra NAP 5 columns were purchased from VWR (Canada). All other reagents and solvents were of the analytical grade and purchased from Sigma Aldrich. Ultrapure water was used throughout the experiments.

**Table 1:** Sequences of the oligonucleotide probes with a specific *Cryptosporidium* 42-nt RNA and non-complementary RNA were used in this study.

Oligonucleotide	Sequence
Oligonucleotide probe A	5′/5ThioMC6-D/TTTTTTTTTTA_15_ATTGTTATTTCTTGTCACTAC-3′
Oligonucleotide probe B	5′/5ThioMC6-D/T_9_TA_14_ATACAAAACCAAAAAGTCCTGT-3′
*Cryptosporidium* 42-nt RNA target	5′-GUAGUGACAAGAAAUAACAAUACAGGACUUUUUGGUUUUGUA-3′
Non-complementary RNA target	5′-CAUCACUGUUCUUUAUUGUUAUGUCCUGAAAAACCAAAACAU-3′

## Instruments

All ultraviolet–visible (UV–vis) spectral measurements were carried out by double beam spectrophotometer, Cary 3500 UV–vis. A 1-cm path length quartz cuvettes were used for all measurements. An Oakton pH meter (Oakton Instruments, pH 510 series) was calibrated against three standard buffer solutions with three different pH values (4.0, 7.0 and 10) and used for the pH determination.

## Experimental methods

### Sensor fabrication

The fabrication of the chip-based colorimetric biosensor was performed as previously described in^76^. All glass slides were cleaned by using piranha solution followed by an oxygen plasma treatment for 10 min. Chromium and gold layers with thicknesses of 50 nm and 250 nm, respectively, were sputtered (Angstrom Engineering) on the cleaned glass slides in an argon atmosphere. Sensing transparent circular zones with a diameter of 3 mm were patterned using the standard lithography process, as described in^3^. A lift-off procedure was followed to create hydrophobic circular barriers around the detection zone. This process has led to circular hydrophobic barriers around the detection spot to control the sample in the sensing area.

### Holder assembly fabrication

**Figure 2:**
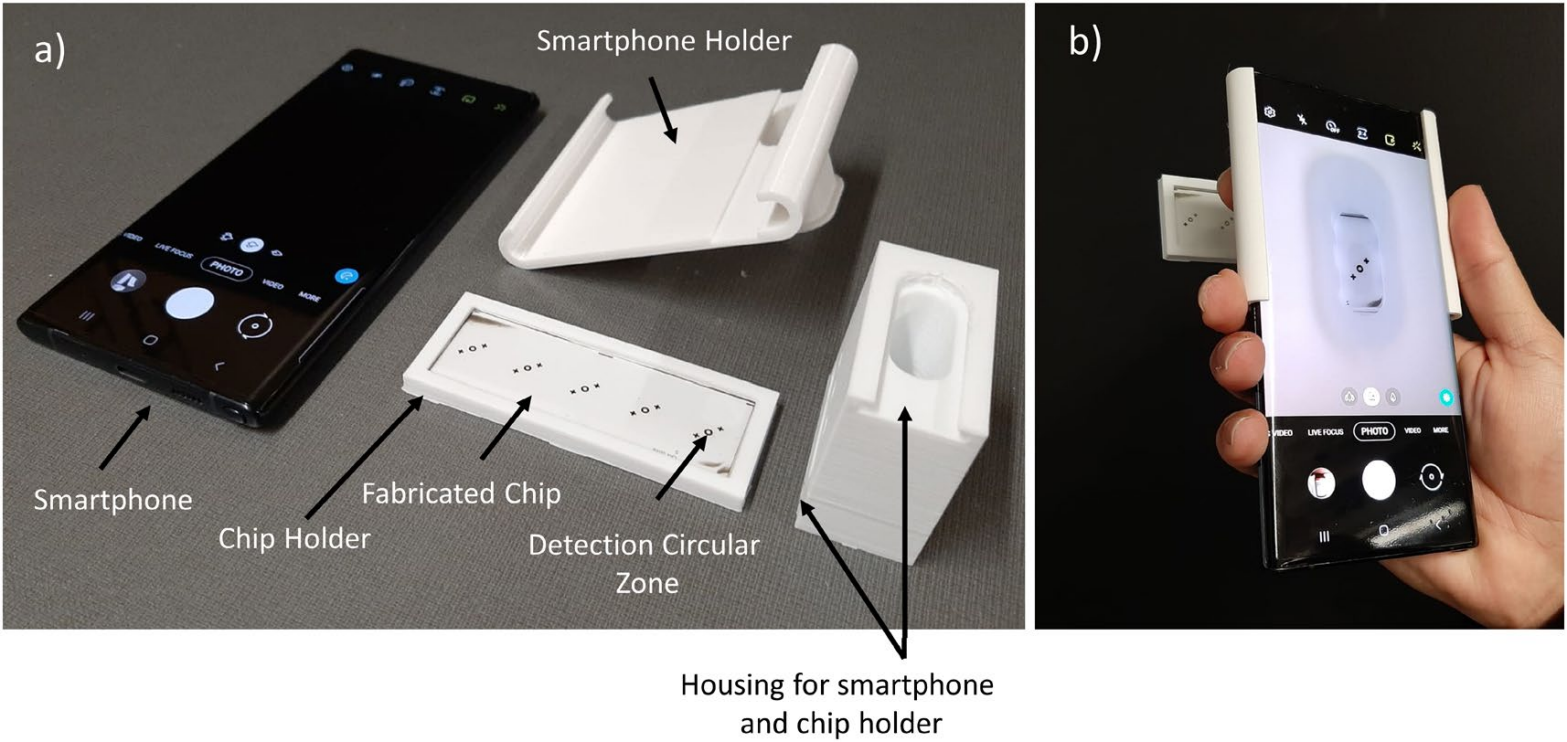
The fabricated chip and 3D portable holder assembly are integrated with a smartphone. (**a**) the major components of the detection system, (**b**) assembled detection system.

The 3D portable holder assembly integrated with a smartphone is shown in Fig. 2. A smartphone camera is used to collect image samples. An open-source application (Color Grab, Loomatix) from the android store is used to analyze the collected images and display the analyte concentration to the user. A battery built in the smartphone is used to supply power. This allows the smartphone to be charged using an inexpensive micro-USB connector, enabling the smartphone to operate on the battery power for several hours^43^. The smartphone touch screen is used to control the detection process. Furthermore, the smartphone's built-in Wi-Fi chip allows a 2.4 GHz wireless network to transfer data for further analysis on an external computer or external storage when required. A three-dimensional-printed holder assembly was designed and fabricated to house all the chips and the smartphone inside a compact unit.

### Conjugation of thiolated oligonucleotide to gold nanoparticles

The conjugation of the two sets of thiolated oligonucleotides (Table 1) to gold nanoparticles was carried out via two parts involving: (1) the reduction of the two sets of thiolated oligonucleotides, and (2) the conjugation of the reduced oligonucleotides to the gold nanoparticles. In the first part, the two sets of lyophilized oligonucleotides were dissolved to a final concentration of 500 µM in ultrapure water. A 50 µL dissolved oligonucleotide was then mixed with 450 µL of 0.15 M sodium phosphate buffer supplemented with 0.1 M DTT. Following this step, the mixture was incubated at room temperature for 2 h to reduce the oligonucleotides. Subsequently, A NAP 5 column operated in water was used to separate the reduced oligonucleotides from trityl-SH and DTT. The final elute from the NAP 5 column was 1 mL in water with an approximate concentration of 25 μM. The final concentration of the elute was measured with UV–vis spectroscopy by measuring the absorbance at 260 nm.

A 1 mL solution of unmodified gold nanoparticles was transferred to a 1.5 mL Eppendorf tube in the second part. Then, 24 µL of the reduced thiolated oligonucleotides (25 μM in water, equivalent to 0.025 nmol/μL) was added to the gold nanoparticles and incubated for 1 h at room temperature. Following this step, 0.1 M sodium phosphate buffer (pH 7.0) was added to the mixture in a stepwise manner (in 10 μL increment with 5 min incubation time in between) until a final concentration of 10 mM sodium phosphate (pH 7.0) was achieved. Subsequently, 1 M NaCl was added in a similar stepwise manner until a final concentration of 100 mM was reached. The mixture was then incubated at 4 °C for 16 h. The above steps minimize the aggregation of the gold nanoparticles by immobilizing the thiolated oligonucleotides properly onto the gold nanoparticles' surface, which improves the overall stability and quality of the conjugation process. The conjugated gold nanoparticles were then centrifuged at 17,000×g for 30 min to pellet the oligonucleotide gold conjugate. Lastly, the supernatant was removed, and the oligonucleotide gold conjugate was resuspended in 200 μL of 10 mM sodium phosphate buffer, 100 mM NaCl and 0.01% (w/v) NaN_3_.

### Sample preparation and colorimetric measurements of *Cryptosporidium* RNA

For the colorimetric detection of *Cryptosporidium* RNA using the developed sensing platform, 3 µL of AuNPs modified with probe A (25 μM), and 3 µL of AuNPs modified with probe B (25 μM) were first added to the sensing zone on the fabricated chip. Next, 5 µL of different concentrations of *Cryptosporidium* RNA (5, 25, 60, 100, 200 and 300 µM) and 4 µL of hybridization buffer (a stock solution of 16% dextran sulfate and 20% formamide) were added to the modified AuNPs. The chip was then incubated at 70 °C for 15 min, followed by incubation at 37 °C for 20 min. Lastly, the chip was inserted in the holder assembly at room temperature. The presence of *Cryptosporidium* RNA in the sample results in a detectable color change after 5 min in the detection zone patterned on a chip, which can be visualized by the naked eye.

For quantitative analysis, the color change is captured by a smartphone camera (Samsung Galaxy Note 10) integrated with a 3D-printed holder assembly under controlled light conditions. A region of interest (ROI) in the captured image was selected and fixed for consistency measurements. An open-source application (Color Grab, Loomatix) from the android application store was installed on the smartphone and used for converting the ROI from the red, green, blue (RGB) color space to the Hue, Saturation, Value (*HSV*) color space. This previous step was performed to achieve a linear relationship between the color components and the color change. Image analysis was performed by calculating the Saturation value of the recorded images' *HSV* coordinates and correlated to the sample's analyte concentration.

For the UV–vis measurements, the same concentrations were used in a 1 mL volume sample. All measurements were performed before and after the incubation with different Cryptosporidium RNA concentrations and were repeated three times to confirm the detection limit and conduct a statistical error analysis.

### Calibration curve, limit of detection (LOD), and selectivity

To assess the developed on-chip colorimetric sensor's applicability, a calibration curve was obtained by plotting A_630_/A_523_ nm absorption ratio and Saturation values of the *HSV* coordinates of the recorded images versus the RNA concentration using the UV–vis and the smartphone, respectively. The limit of detection (LOD) was calculated using the following expression: LOD = 3SD_b_/*m,* where SD_b_ is the standard deviation of the blank and *m* is the slope of the calibration curve^77,78^.

To evaluate the developed sensing platform's selectivity, aqueous solutions spiked with a non-complementary RNA consisting of 42 nucleotides (random sequence RNA) were tested and used as the control (see Table 1).

## Results and discussion

### Evaluation of AuNPs conjugation using UV–visible spectroscopy

**Figure 3:**
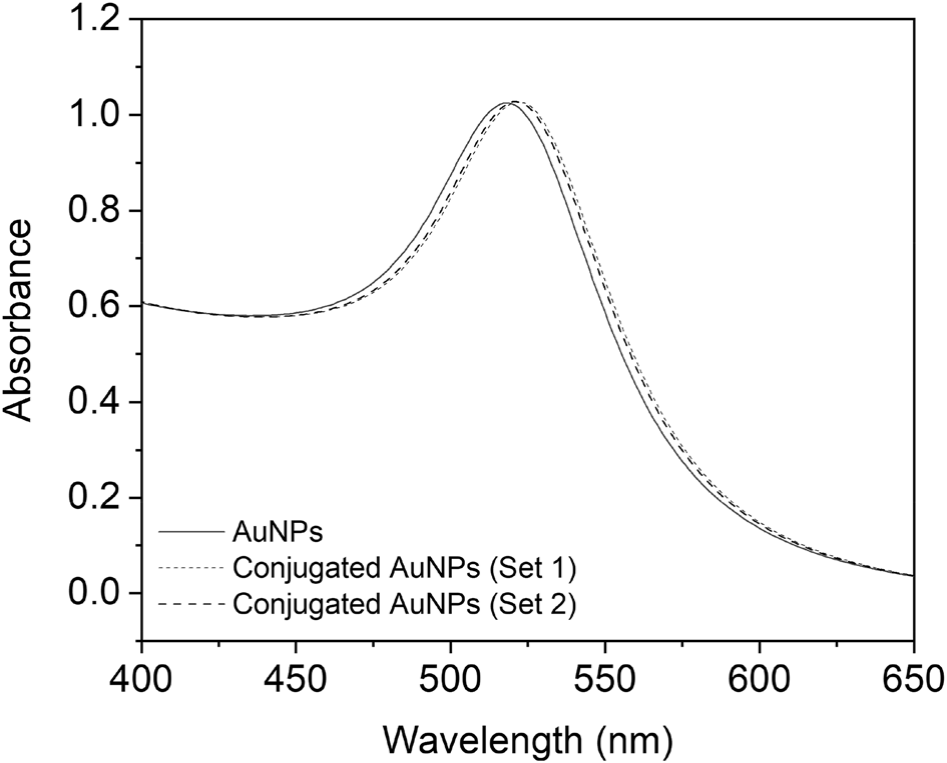
UV–vis absorption spectra of gold nanoparticles before and after conjugation with probe A (25 μM (set 1)) and probe B (25 μM (set 2)).

To confirm the conjugation of AuNPs with the oligonucleotides, UV–vis spectra were recorded for AuNPs colloid (20 nm) before and after conjugation (Fig. 3). Gold nanoparticles colloid (with a diameter of 20 nm) exhibits an extinction maximum at wavelength 520 (Fig. 3). After conjugating the AuNPs with oligonucleotides, a slight shift of 3 nm in the absorption wavelength was observed. This slight shift confirms the conjugation of AuNPs.

### Detection of *Cryptosporidium* RNA using UV–vis spectroscopy

**Figure 4:**
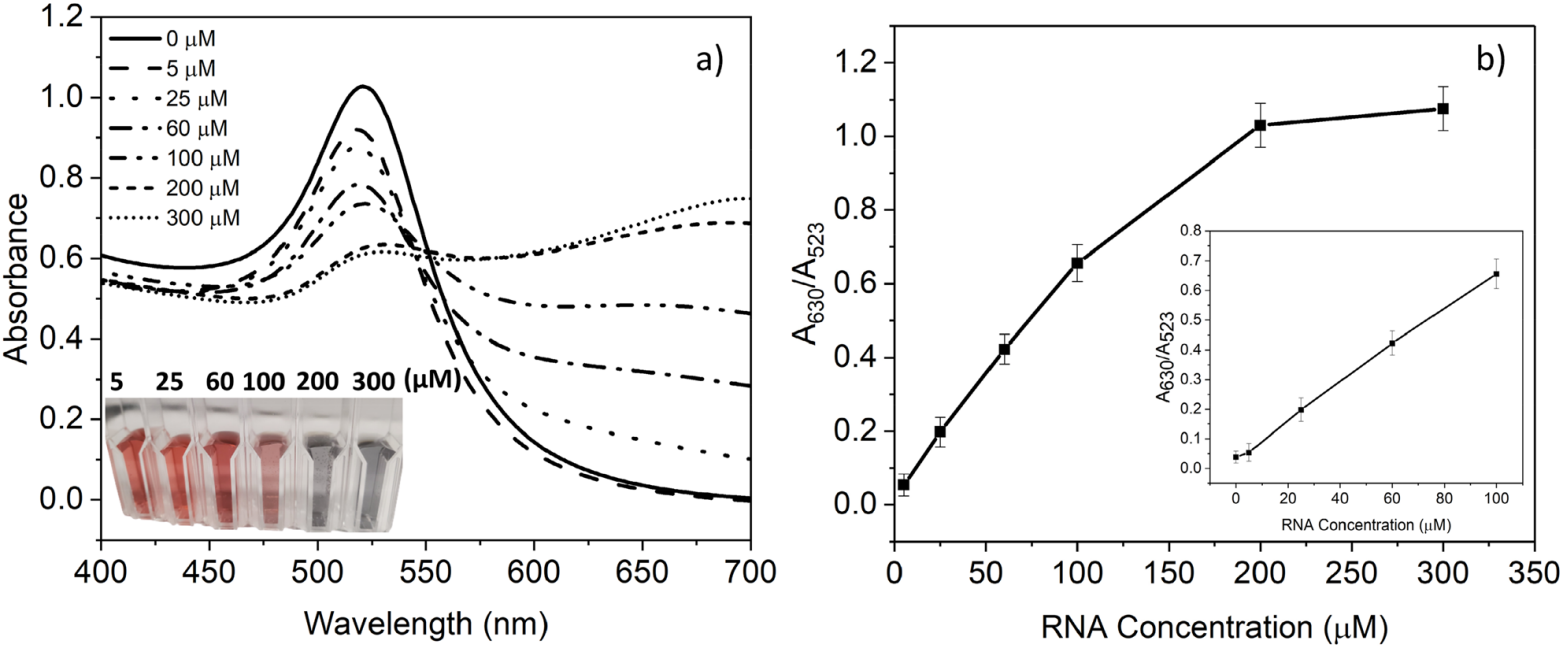
Detection of *Cryptosporidium* RNA using UV–visible spectroscopy. (**a**) UV–vis absorbance spectra of conjugated AuNPs after adding *Cryptosporidium* RNA at different concentrations (5, 25, 60, 100, 200 and 300 µM) to the conjugated gold nanoparticles with probe A (25 μM) and probe B (25 μM). Inset images show the color of the modified AuNPs in the presence of different concentrations of *Cryptosporidium* RNA. (**b**) calibration curve plot of the A_630_/A_523_ ratio versus different concentrations of *Cryptosporidium* RNA. The inset plot shows the linear range for different concentrations of *Cryptosporidium* RNA.

The colorimetric assay was first tested with different *Cryptosporidium* RNA concentrations, as described in “Sample preparation and colorimetric measurements of *Cryptosporidium* RNA” section, and verified using UV–vis spectroscopy. The UV–vis spectroscopy results in Fig. 4a) show that the presence of different analyte concentrations in the sample results in a decrease in the absorption peak at 523 nm, while there is a gradual increase in the absorption peak of 630 nm. Hence, the A_630_/A_523_ ratio was used in this study to investigate the aggregation state of the modified AuNPs. Figure 4b) shows that increasing the target analyte's concentration from 5 µM to 300 µM has led to a gradual increase in the A_630_/A_523_ ratio, and a noticeable color change of AuNPs from red to blue was observed.

Furthermore, the color change can be visualized at high concentrations of the target analyte (> 25 µM) (see the inset in Fig. 4a)). The A630/A523 ratio plotted versus the RNA concentration in the sample exhibited an excellent linear range between 5 µM and 100 µM with a detection limit of approximately 5 µM. The detection limit (calculated as described in “Calibration curve, limit of detection (LOD), and selectivity” section) was determined from the calibration curve created by plotting the 630/523 nm absorption ratio, demonstrating the degree of AuNPs aggregation versus the analyte concentration. Although the apparent detection limit visualized by the naked eye was 25 µM (see the inset in Fig. 4a)), quantitative analysis needs to be performed relative to the blank color. Otherwise, misanalysing under different environmental conditions can occur.

### Detection of *Cryptosporidium* RNA using on-chip biosensor

**Figure 5:**
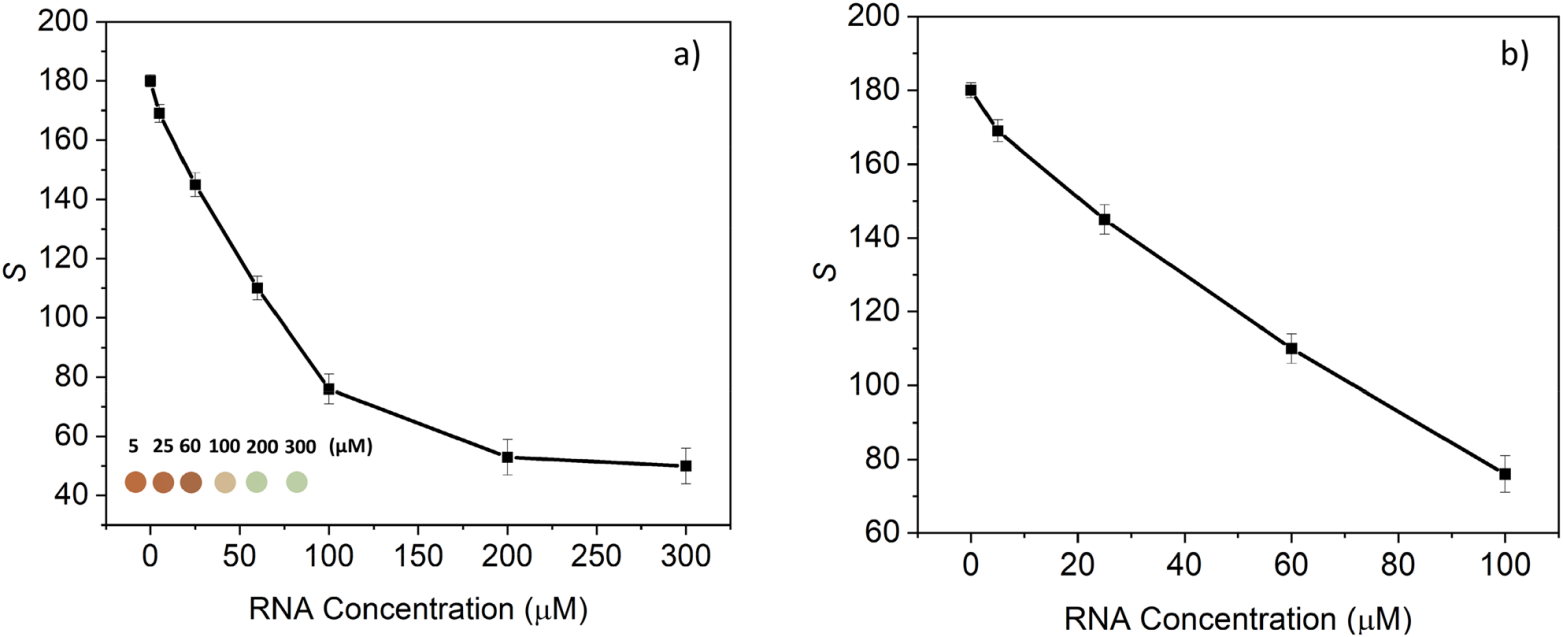
Detection of *Cryptosporidium* RNA using the developed sensing platform. (**a**) calibration curve plot of the Saturation value (S) of the *HSV* coordinates of the recorded images versus different *Cryptosporidium* RNA concentrations interacting with the conjugated gold nanoparticles with probe A (25 μM) and probe B (25 μM). (**b**) the linear range for different concentrations of *Cryptosporidium* RNA.

The sensor response from different concentrations of *Cryptosporidium* RNA is presented in Fig. 5. The colorimetric results show that increasing the analyte concentration from 5 µM to 300 µM results in a decrease in the Saturation value (*S*) of the *HSV* coordinates of the recorded images and a distinct color change (see the inset in Fig. 5a)). We found that the *S* value is more reliable and accurate than the *H* and *V* values for monitoring the sample's RNA concentration. The sensor showed a wide linear response between 5 µM and 100 µM with a detection limit of approximately 5 µM (see Fig. 5b)). The results obtained by the developed sensing platform show a good agreement with the results obtained by the conventional method (UV–vis spectroscopy). However, the sensing platform results are more easily differentiable (see the inset in Fig. 5a)).

Additionally, the sample volume used with the on-chip method was 15 µL, making the proposed sensing platform safer and less expensive than the UV–vis spectroscopy that requires a larger sample volume (e.g., 1 mL). Furthermore, small volumes result in a shorter analysis time so that the total analysis time can be reduced from hours to 30 min. Moreover, multiple samples from different contaminated sites can be analyzed at once, which will further shorten the analysis time and result in a large-scale screening process.

The detection limit achieved was high (5 µM) compared to those reported in^79^. However, unlike our sensing platform, in^79^, DNA amplification was conducted to enhance the sensitivity. This has resulted in increasing the cost, analysis time and hindered portability and thus on-site detection. In our sensing platform, DNA oligonucleotides were used and the capture probe for the detection of RNA. This has increased the number of base-pairs mismatch between DNA and RNA and thus incomplete hybridization, which results in less sensitivity and detection limit. The use of RNA oligoprobes (capture probe) is expected to enhance the detection limit and sensitivity by 10 orders of magnitude. This is due to the high degree of stability of RNA/RNA hybrid duplexes compared to RNA/DNA ones^80^.

### Selectivity of colorimetric assay towards *Cryptosporidium* RNA

**Figure 6:**
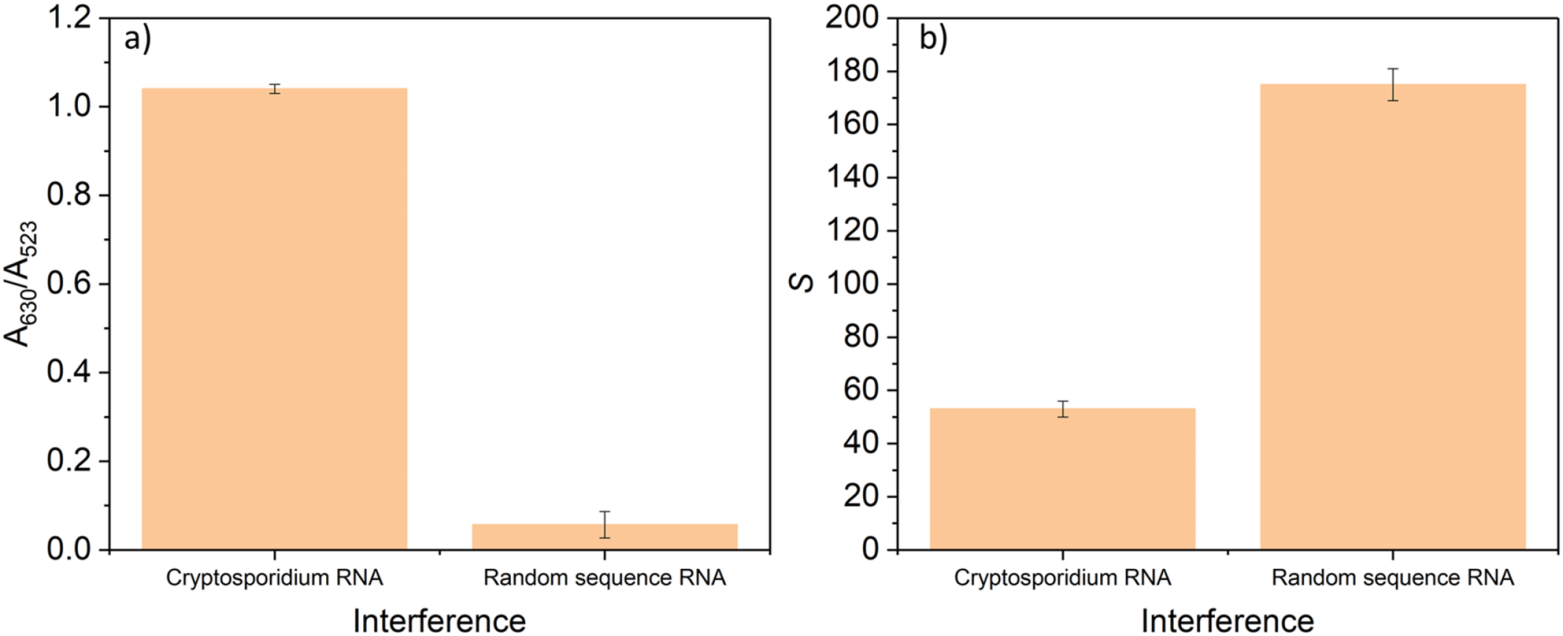
Selectivity results were obtained by (**a**) UV–vis absorption spectra and (**b**) the developed sensing platform in the presence of the analyte of interest and the random sequence RNA. All measurements were performed using the same conditions and a final concentration of 200 µM of the target analyte and the random sequence.

To investigate the selectivity of the developed colorimetric assay, the degree of the color change of the modified AuNPs towards the analyte of interest and a non-complementary sequence RNA consists of 42 nucleotides (random sequence RNA) were recorded by both UV–vis spectroscopy (Fig. 6a)) and the developed sensing platform (Fig. 6b)). In this experiment, the concertation of the target analyte and the random sequence was 200 µM. A high concentration of *Cryptosporidium* RNA and the random sequence RNA was chosen to investigate the effect of other random bacterial sequences RNA in high-contaminated samples. The results show that a high concentration of a random sequence RNA does not induce any noticeable color change (minor changes of A_630_/A_523_ ratio in Fig. 6a)) to the modified AuNPs. In contrast, with the addition of 200 µM of *Cryptosporidium* RNA, a significant increase of A_630_/A_523_ was observed. In the case of the developed sensing platform (see Fig. 6b)), a considerable decrease in the *S* value from 175 (random sequence RNA, which approximately equals to the *S* value of the blank) to 53 (*Cryptosporidium* RNA). The results confirm the high selectivity of the developed colorimetric assay and the sensing platform to detect *Cryptosporidium* RNA in complex samples.

### Data validation

Additional experiments were performed to investigate the repeatability, practicality, accuracy and reliability of the developed sensing platform. Five samples with different concentrations (5, 10, 15, 20 and 25 µM) were prepared as described in “Sample preparation and colorimetric measurements of *Cryptosporidium* RNA” section. All measurements were performed under the same conditions as those performed for the calibration measurements. Table 2 summarizes a comparison between the prepared sample concentrations and those obtained by the developed sensing platform.

**Table 2:** Application of the developed sensing platform for the detection of *Cryptosporidium* RNA samples spiked with different concentrations of *Cryptosporidium* RNA.

RNA concentration in the sample (µM)	RNA concentration measured by the developed sensing platform (µM)	Error (%)
5.0	6.0	20.0
10.0	10.3	3.0
15.0	15.5	3.3
20.0	20.7	3.5
25.0	24.3	2.8

In general, the obtained results are in good agreement, with maximum errors occurring at the lowest concentration (5 µM).

Table 3 shows a comparison of methods reported in the literature to date for the detection of *Cryptosporidium* with the developed sensing platform in terms of detection principle, advantages, and disadvantages.

**Table 3:** Comparative evaluation of the conventional and state-of-the-art methods previously developed for the detection of *Cryptosporidium* with the sensor developed in this research.

Technique	Detection principle	Advantages	Disadvantages	Refs.
Microscopy	This technique requires fluorescence labels. An appropriate fluorescence microscope is then used to count the labeled oocysts	Cost-effective Widely-available	Require time-consuming labelling steps and expensive labels Low sensitivity Time-consuming and require trained personnel Unsuitable for in-field detection	^81,82,83,84^
Immunological Techniques (ELISA)	This technique requires labelling the analyte of interest or the capturing probe with an appropriate colorimetric or fluorescence label. The presence of the target analyte is then detected by measuring the change in the colorimetric or fluorescence signal	Excellent sensitivity Commercially available Suitable for analyzing a large number of samples Can be automated	Expensive, particularly for resource-poor countries Time consuming Require many preparation steps Require complicated labeling steps, expensive labels and expensive instrumentations Require trained personnel and technically demanding	^12,85,86^
Molecular techniques	These techniques rely mainly on using PCR amplification of the pathogen DNA. PCR detection involves the extraction of the DNA from *Cryptosporidium* and the amplification of a specific sequence of that DNA. The amplified region is detected using an appropriate technique such as agarose gel electrophoresis	Excellent sensitivity and accuracy Able to identify the pathogen species	Technically demanding Require trained personnel for DNA extraction and amplification Time consuming Require high concentration of the target DNA for good amplification Different primers are required for different pathogens Slight change in primers leads to lower sensitivity, false-positive, and false-negative results Suffers from interference from PCR inhibitors in environmental samples Unsuitable of on-site detection	^87,88,89,90,91^
Capacitive	This technique involves measuring change in capacitance at a specific frequency as a result of biorecognition event	Label/PCR free Suitable for on-site detection Sensitive	Frequency-dependent Detrimentally affected by environmental conditions Any change in temperature and moisture leads to false-positive and false-negative results	^2,3,92^
Electrochemical	This technique involves measuring change in current or resistance-charge transfer as a result of the biorecognition event	Sensitive and fast Label/PCR free Cost-effective Low power requirement Easy to miniaturize and robust Ability to be used with matrix samples contaminated with optically and fluorescence absorbing molecules Can be used for on-site detection	Sensitive to environmental conditions Low-shelf life Less-sensitive than the conventional method’s such as PCR	^93,94,95,96,97^
Surface plasmon resonance (SPR)	This method is based on measuring change in refractive index due to the interaction between *Cryptosporidium* and the immobilized biological recognition element	Sensitive and suitable for real-time measurements Label-free The sensor can be regenerated Can be used to detect complex sample with no need for purification prior to detection Reproducible Can be miniaturized	Expensive Suffer from non-specific binding Low mass transport	^98,99,100,101,102^
Colorimetric (This research)	It involves measuring the color change as a result of the biorecognition event	Affordable and accessible Easy to use, to implement, and to operate Have a low cost of distribution Cost-effective and can be miniaturized Rapid The color change can be visualized qualitatively by the naked eye (no optical instrument needed) or measured quantitively by an optical instrument such as a smartphone Can be used for the detection of a wide variety of analytes Can be integrated with smartphones technology for POC diagnostics Suitable for resource-limited countries	Suffer from limited environmental control Can be negatively impacted by ambient light or vibrations (The solution to overcoming these challenges in the future are discussed in the conclusion section)	^103,104,105,106^

### Conclusions

A simple and cost-effective colorimetric assay combining the chemical and photoelectric properties of AuNPs on a chip with 3D-printed holder assembly integrated with a smartphone was developed for the sensitive, label-free and on-site detection of *Cryptosporidium* specific RNA*.* This sensing platform eliminated the need for PCR amplification, well-trained personnel and bulky instruments. Two sets of AuNPs modified with oligonucleotides capturing probes specific and complementary to adjacent sequences on *Cryptosporidium* rRNA were used to enhance the specificity and selectivity towards the analyte of interest to reduce the inference from undesired substances in the sample. After assessing the colorimetric assay using UV–vis spectroscopy, the assay was tested using the developed sensing platform to confirm the applicability, reproducibility and reliability of the sensing platform for the quantitative detection of *Cryptosporidium.*

Under optimized conditions, results obtained from the UV–vis spectroscopy and the developed sensing platform showed a linear detection range between 5 and 100 µM with a detection limit of 5 µM. The detection limit obtained by the developed sensing platform was similar to the detection limit obtained by UV–vis spectroscopy without using bulky and advanced laboratory equipment. The total turnaround analysis time using the developed sensing platform was reduced from hours to 30 min. Even when a high concentration of random sequences RNA in spiked samples were tested with the developed sensing assay, the developed sensing platform showed high selectivity and specificity towards *Cryptosporidium* RNA. Furthermore, the sample volume used for measurements using the developed sensing platform was 15 µL, which reduces the sample volume and cost required for a single measurement required for analysis compared to the conventional method. In addition to the direct measurement of *Cryptosporidium* RNA without amplification, this sensing platform enables a simple way to screen multiple samples for *Cryptosporidium* from different contaminated sites and quantitatively determine the concentration of *Cryptosporidium* in the sample.

This smartphone-integrated sensing platform is a steppingstone towards creating ubiquitous and accessible pathogen detection technologies that could potentially be used in resource-limited settings, however, there are some outstanding challenges that need to be met before a field-ready technology is accomplished. One limitation of this technology is that it suffers from limited environmental control when used in open environments where ambient light or vibrations can negatively impact the sensing performance. Overcoming this challenge requires constructing the 3D attachment or enclosure with all the necessary hardware to perform measurements and then connect the attachment through Bluetooth or wire to the smartphone. This will reduce the interference from ambient light and vibrations and enable better control and stability of the attachment. Another challenge is lowering the detection limit below 5 µM of *Cryptosporidium* RNA. The developed sensing platform shows maximum errors at low RNA concentrations. Future research efforts are required to characterize the effect of AuNPs size and RNA-induced aggregation in order to enhance the detection limit. Future studies are also required to study the replacement of the DNA oligonucleotides with RNA oligoprobes to increase the degree of stability of RNA/RNA hybrid duplexes compared to the RNA/DNA ones^80^ as well as testing the sensor with other potential interferants. Multidisciplinary research efforts are required to design and test real-time and high-throughput systems for detecting *Cryptosporidium* spp. in the water supply chains with high sensitivity and reproducibility in order to harmonize global efforts to monitor and prevent waterborne infectious diseases using such technologies.
